# Genetic ancestry and the SNP RS4988235 of lactose tolerance in the population of Lima

**DOI:** 10.17843/rpmesp.2025.422.14223

**Published:** 2025-06-02

**Authors:** Sergio Vladimir Flores, Ángel Roco-Videla, Román M. Montaña

**Affiliations:** 1 Arturo Prat University, Iquique, Chile. Arturo Prat University Arturo Prat University Iquique Chile; 2 Catholic University of the Holy Conception, Chile. Catholic University of the Holy Conception Catholic University of the Holy Conception Concepción Chile; 3 Faculty of Health Sciences, Autonomous University of Chile, Santiago, Chile. Autonomous University of Chile Faculty of Health Sciences Autonomous University of Chile Santiago Chile

To the Editor, The rs4988235 polymorphism, located in the regulatory region of the MCM6 gene, influences the expression of the LCT gene, which is responsible for lactase production, and is associated with lactose intolerance. This polymorphism shows population variability, with the A allele (associated with lactose tolerance) prevalent in northern Europe, while the G allele (associated with intolerance) is more common in Asia and Africa ^1^^,^^2^. In Latin American populations, the frequency of these alleles varies considerably due to genetic mixing and migratory history ^3^. Integrating the proportion of genetic ancestry into the analysis of tolerance or intolerance alleles is relevant for understanding the distribution of lactose intolerance in these populations. Therefore, the objective of this preliminary study was to evaluate the relationship between the proportions of genetic ancestry (European, Native American, and African) and the genotypes of the SNP (Single Nucleotide Polymorphism) rs4988235 associated with lactose tolerance or intolerance in the population of Lima, Peru.

The genotypes for rs4988235 were obtained from the public database of the 1000 Genomes Project (https://www.internationalgenome.org), selecting the sample from Lima (n=85). To estimate genetic ancestry proportions, 446 SNPs from a panel of informative ancestry markers were used, as described by Galanter *et al*. ^4^, considering five macro-populations: African, East Asian, South Asian, European, and Latin American.

The proportions of genetic ancestry were modeled using the STRUCTURE program ^5^^,^^6^. The Shapiro-Wilk test confirmed that ancestry distributions did not follow a normal distribution (p<0.05). The nonparametric Kruskal-Wallis and Mann-Whitney U tests were applied to evaluate the association between genetic ancestry proportions (independent variables) and lactose intolerance genotypes (dependent variables).

**Figure 1:**
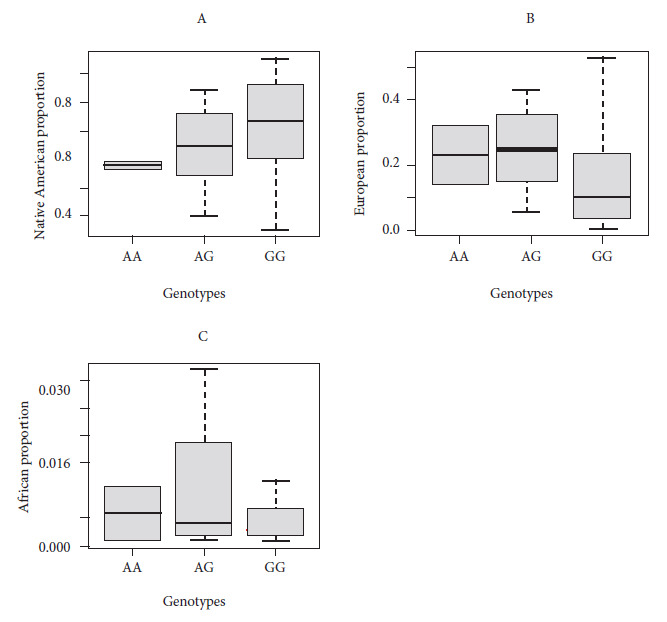
Genetic ancestry and rs4988235 in the LCT gene in the Lima population. Figures A, B, and C show the proportions of genetic ancestry according to the genotypes of the rs4988235 polymorphism in the LCT gene.

The Kruskal-Wallis test showed differences in the proportion of European ancestry among the three genotypes (AA, N= 9; AG, N = 34 and GG, N= 42) of lactose tolerance and intolerance (p=0.018), but no significant differences were found for Native American (p=0.089) or African (p=0.772) ancestry. Post hoc analysis with the Mann-Whitney U test revealed no significant differences between genotypes for the proportion of Native American and European ancestry (p>0.05). Figure 1 shows the distribution of genetic ancestries according to LCT genotypes.

This study has some limitations. First, the sample is limited to the population of Lima, Peru, so the conclusions cannot be extrapolated to other populations. In addition, the estimation of genetic ancestry is based on a specific panel of SNPs, which may not reflect all relevant genetic variants. Given the exploratory nature of the study and the use of a public database, the statistical power was not calculated.

In conclusion, our results suggest that the proportion of European ancestry is associated with lactose tolerance. These findings highlight the importance of considering the genetic heterogeneity of populations in Latin America to understand the population distribution of biomedical phenotypes of interest.
