# Supplementary material for: Genetic ancestry and the SNP RS4988235 of lactose tolerance in the population of Lima
Source: Rev Peru Med Exp Salud Publica. 2025 Jun 2;42(2):225–6. doi: 10.17843/rpmesp.2025.422.14223 (PMC12377878; doi:10.17843/rpmesp.2025.422.14223)
Supplement: Translation

# Translation: Ancestría genética y el SNP RS4988235 de tolerancia a la lactosa en población de Lima

**Authors:** Sergio Vladimir Flores, Ángel Roco-Videla, Román M. Montaña

**Affiliations:** 1 Universidad Arturo Prat, Iquique, Chile.; 2 Universidad Católica de la Santísima Concepción, Concepción, Chile.; 3 Facultad de Ciencias de la Salud, Universidad Autónoma de Chile, Santiago, Chile.

Sr. Editor. El polimorfismo rs4988235, ubicado en la región reguladora del gen MCM6, influye en la expresión del gen LCT, responsable de la producción de lactasa, y está asociado con la intolerancia a la lactosa. Este polimorfismo presenta variabilidad poblacional, siendo el alelo A (asociado a la tolerancia a la lactosa) prevalente en el norte de Europa, mientras que el alelo G (asociado a la intolerancia) es más común en Asia y África ^1^^,^^2^. En las poblaciones de América Latina, la frecuencia de estos alelos varía considerablemente debido a la mezcla genética y la historia migratoria ^3^. Integrar la proporción de ancestría genética en el análisis de los alelos de tolerancia o intolerancia es relevante para comprender la distribución de la intolerancia a la lactosa en estas poblaciones. Por lo tanto, el objetivo de este estudio preliminar es evaluar la relación entre las proporciones de ancestría genética (europea, indígena americana y africana) y los genotipos del SNP (Single Nucleotide Polimorphism) rs4988235 asociado a la tolerancia o intolerancia a la lactosa en la población de Lima, Perú.

Los genotipos para rs4988235 se obtuvieron de la base de datos pública del Proyecto 1000 Genomas (https://www.internationalgenome.org), seleccionando la muestra de Lima (n=85). Para estimar las proporciones de ancestría genética, se utilizaron 446 SNP de un panel de marcadores informativos de ancestría, según lo descrito por Galanter *et al*. ^4^, considerando cinco macropoblaciones: africana, asiática oriental, asiática meridional, europea y latinoamericana.

Las proporciones de ancestría genética se modelaron utilizando el programa STRUCTURE ^5^^,^^6^. La prueba de Shapiro-Wilk confirmó que las distribuciones de ancestría no seguían una distribución normal (p<0,05). Se aplicaron las pruebas no paramétricas de Kruskal-Wallis y U de Mann-Whitney para evaluar la asociación entre las proporciones de ancestría genética (variables independientes) y los genotipos de intolerancia a la lactosa (variables dependientes).

La prueba de Kruskal-Wallis mostró diferencias en la proporción de ancestría europea entre los tres genotipos (AA, N= 9; AG, N = 34 y GG, N= 42) de tolerancia e intolerancia a la lactosa (p=0,018), pero no se observaron diferencias significativas para las ancestrías indígena americana (p=0,089) ni africana (p=0,772). El análisis post hoc con la prueba U de Mann-Whitney no reveló diferencias significativas entre los genotipos para la proporción de ancestría indígena americana y europea (p>0,05). La figura 1 muestra la distribución de las ancestrías genéticas según los genotipos de LCT.

**Figura 1:** Ancestría genética y rs4988235 en el gen LCT en la población de Lima. Las figuras A, B y C muestran las proporciones de ancestría genética según los genotipos del polimorfismo rs4988235 en el gen LCT.

Este estudio presenta algunas limitaciones. En primer lugar, la muestra se limita a la población de Lima, Perú, por lo que las conclusiones no pueden extrapolarse a otras poblaciones. Además, la estimación de la ancestría genética se basa en un panel específico de SNP, lo que podría no reflejar todas las variantes genéticas relevantes. Dado el carácter exploratorio del estudio y el uso de una base de datos pública, no se realizó un cálculo de potencia estadística.

En conclusión, nuestros resultados sugieren que la proporción de ancestría europea se asocia con la tolerancia a la lactosa. Estos hallazgos destacan la importancia de considerar la heterogeneidad genética de las poblaciones en América Latina para comprender la distribución poblacional de los fenotipos biomédicos de interés.
